# 

**Published:** 1918-08-31

**Authors:** 


August 31st, 1918.] [Price Twopence

### The Hospital

The Workers' Newspaper of Administrative Medicine and Institutional Life, Administration, National Insurance and Health.

CONTENTS.

Maternity and Child Welfare ... ... ... ... 463

Arterio-Sclerosis and Blood Pressure ... ... 464

Hospital and Institutional News ... ... ... 465

An Instruction Mr. Churchill Should Issue—More Beds for Civilians at Boscombe—Autographs and Charity—Hospital Work in Palestine—Unwilling Volunteers—A Fine Example of Continuity—A New Cottage Hospital—A Woman Hospital Medical Superintendent—Criticism of Provision for Treatment of Tuberculosis—Refreshments for Out-patients—Physical Clinics on the Increase—New Zealand Hospital Finance—Suggestions for Retrenchment—To Paint or Not to Paint—The Retention of a Sufficient Staff—The Downs Sanatorium—A Lucky Surgical Instrument Maker—A Collective Fee for a Batch of School Cases?—Commandeering Hospitals for Infectious Disease—Salaries and Competition—A Remarkable Body of Nurses—This Week's Drug Market.

Papers on Madness ... ... ... ... ... 469

III. The Origin of the Act of 1890.

The Simulation of Disease ... ... ... ... 471

II. Some Medical Aspects of Malingering.

Australian Notes ... ... ... ... ... ... 473

CONTENTS.

A Nurses' Charter of Liberty ... ... ... ... 474

The Cry for Freedom.

The Matrons' and Sisters' Department ... ... 475

Nursing Progress and its Developments: The Local Executive Committee.

Round the Hospitals ... ... ... ... ... 475

New Premises for the College—A London Local Centre—Mesopotamian and S.W. Africa Honours—Institution Nurses and the Vote—Dismissal "Without Reason Assigned"—Women's Part in Liberating the World.

The Woman Worker's World ... ... ... 479

Passing Events and Topics ... ... ... ... 479

Maternity and Child Welfare Act ... ... ... 480

A Summary of the L.G.B. Circular.

A Boycotted Hospital ... ... ... ... ... 480

Matrons' and Sisters' Appointments ... ... ... vi

The Editor's Letter-Box ... ... ... ... vi

Holidays for Mothers.

Institutional Worker ... ... ... ... ... I

Bedroom Suppers for Nurses Off Duty: Answer to July Question.

Lectures on Infant Care.

